# Supplementary material for: Robust neuromorphic coupled oscillators for adaptive pacemakers
Source: Sci Rep. 2021 Sep 10;11:18073. doi: 10.1038/s41598-021-97314-3 (PMC8433448; doi:10.1038/s41598-021-97314-3)
Supplement: Supplementary file 1 — Supplementary Information. [file 41598_2021_97314_MOESM1_ESM.pdf]

Robust neuromorphic coupled oscillators for adaptive pacemakers

Renate Krause[1,*], Joanne J.A. van Bavel[2], Chenxi Wu[1], Marc A. Vos[2], Alain Nogaret[3], and Giacomo Indiveri[1]

[1]Institute of Neuroinformatics, University of Zurich and ETH Zurich, Zurich, Switzerland
[2]Department of Medical Physiology, Division Heart & Lungs, University Medical Center Utrecht, Utrecht, The Netherlands
[3]Department of Physics, University of Bath Claverton Down, Bath, UK
*rekrau@ini.uzh.ch

Supplementary Information

Neuron and synapse model on neuromorphic processor

The equations that describe the behaviour of the neuron and synapse circuits have been derived from the analog circuits[1]. In particular, the equation that describes the subthreshold current I_{mem} representing the membrane potential of the neurons on the DYNAP-SE board is, to first order approximation:

$$\tau \frac{d}{dt} I_{mem} + I_{mem} = I_{mem_\infty} - I_{ahp} + f(I_{mem}) \quad (1)$$

$$\tau_{ahp} \frac{d}{dt} I_{ahp} + I_{ahp} = I_{ahp_\infty} \delta(t) \quad (2)$$

where I_{ahp} is the after-hyperpolarizing current that implements the neuron's spike-frequency adaptation mechanism, τ and τ_{ahp} are the neuron's and the adaptation time constants respectively, $\delta(t)$ is the impulse function that is 1 when the neuron spikes and 0 otherwise. The term $f(I_{mem})$ is an exponential function with positive exponent (see[1] for details). As a consequence, the silicon neuron circuit implements an "adaptive exponential integrate and fire" (AdExp) neuron model[2].

The other parameters in the equation above are:

$$\tau \triangleq \frac{C_{mem} U_T}{\kappa I_\tau} \qquad \tau_{ahp} \triangleq \frac{C_p U_T}{\kappa I_{\tau_{ahp}}} \quad (3)$$

$$I_{mem_\infty} \triangleq \frac{I_g}{I_\tau}(I_{in} - I_{ahp} - I_\tau) \qquad I_{ahp_\infty} \triangleq \frac{I_{g_{ahp}}}{I_{\tau_{ahp}}} I_{Ca} \quad (4)$$

where C_{mem} and C_p are the capacitors used in the circuit to store the membrane potential variable and the spike-frequency adaptation one. The term U_T represents the thermal voltage and κ the subthreshold slope factor. The currents I_τ and $I_{\tau_{ahp}}$ are hyper-parameters that can be used to set the corresponding time constants, and I_g and $I_{g_{ahp}}$ are extra free parameters that can be sued to set global gain terms.

Similarly, to first order approximation, the synaptic input currents I_{syn} that are being summed and sent in input to the neuron (as I_{in} in Eq. 4) can be described as:

$$\tau \frac{d}{dt} I_{syn} + I_{syn} = \frac{I_g I_w}{I_\tau} \quad (5)$$

where I_w is the synaptic weight current, I_g a global synaptic scaling term common to all afferent synapses to the same neuron, and I_τ the current used to set the synapse time constant τ, as defined for the neuron.

References

1. Chicca, E., Stefanini, F., Bartolozzi, C. & Indiveri, G. Neuromorphic electronic circuits for building autonomous cognitive systems. *Proc. IEEE* **102**, 1367–1388 (2014).
2. Brette, R. & Gerstner, W. Adaptive exponential integrate-and-fire model as an effective description of neuronal activity. *J. neurophysiology* **94**, 3637–3642 (2005).
